# Microglia regulate adult neurogenesis via interleukin-6 *trans*-signaling triggered by apoptotic progenitors

**DOI:** 10.21203/rs.3.rs-9619714/v1

**Published:** 2026-06-04

**Authors:** Rudolph Tanzi, Ryan Castro, Evan Gavrilles, Se Hoon Choi

**Affiliations:** Massachusetts General Hospital; Massachusetts General Hospital; Massachusetts General Hospital; Massachusetts General Hospital, Harvard Medical School

## Abstract

In the adult hippocampus, neural progenitor cells (NPCs) proliferate before undergoing differentiation, maturation, and incorporation into the hippocampal neurocircuitry, where they contribute to diverse learning and memory processes that can be perturbed by injury, aging, and disease (1–10). Recent advances have identified microglia and interleukin-6 (IL-6) as regulators of adult hippocampal neurogenesis (AHN) (11). Despite these findings, the mechanism by which IL-6 signaling or microglia regulate neurogenesis has remained unclear. Here, we show that IL-6 trans signaling is triggered by microglial IL-6R shedding during efferocytosis, and that this mediates the transition from proliferation to neuronal differentiation in neighboring, healthy NPCs. We found that proliferating NPCs secrete IL-6 and that apoptotic NPCs are commonly found within clusters of proliferating NPCs. Next, we show that efferocytosis of apoptotic NPCs causes IL-6 receptor shedding by microglia and that IL-6 trans activation of NPCs leads to neuronal differentiation and maturation. Finally, we generated transgenic mice lacking IL-6R exclusively in microglia and found impaired neuronal maturation in the adult hippocampus and deficits in learning and memory in these mice. Our results reveal a molecular mechanism by which microglia regulate adult neurogenesis and contextualize myriad separate investigations into the role of microglia and IL-6 in neurogenesis. Our results position microglia not merely as passive responders to cell death but as active regulators of lineage specification and progenitor cell maturation within the neurogenic niche. The IL-6 trans signaling axis appears to function as a temporally gated checkpoint that coordinates niche refinement—balancing expansion with maturation and synchronizing neuronal development with microglial activation and quiescence. These results could be utilized to develop treatments in pathological contexts characterized by deficits in neurogenesis, such as Alzheimer’s disease.

## Introduction

In the dentate gyrus (DG) of the adult hippocampus, neural progenitor cells (NPCs) proliferate to generate new neurons during adult hippocampal neurogenesis (AHN) (1–4). AHN has been demonstrated to underlie critical aspects of learning and memory, and is severely disrupted in Alzheimer’s disease (AD) (5–10). Recent work shows that microglia can regulate neurogenesis through unidentified signals within their phagocytic secretome (11). The phagocytosis of newborn cells in the subgranular zone (SGZ) of the DG by microglia occurs primarily during the transition from amplifying NPCs to neuroblasts (12). Among the myriad factors secreted by phagocytic microglia is interleukin-6 (IL-6) (11).

Both IL-6 classic- and *trans*-signaling have been implicated in the regulation of neurogenesis, but evidence for the specific roles that these two signaling modalities play in the regulation of microglia and NPCs is mixed (13–22). Evidence indicates a context dependent, pleiotropic role for IL-6 in microglia, but the specific effect of IL-6 signaling events on microglia are still unclear (23–24). Similarly, IL-6 has been implicated as a regulator of embryonic NPCs, but its effect on adult NPCs and the actual mechanism by which this regulation is achieved remain unclear (15,18–19). Here, we identify the cell types in the SGZ that express IL-6 and IL-6R, assess the effects of IL-6 classic- and *trans*-signaling on microglia and NPCs, identify physiologically relevant mechanisms of IL-6R shedding by microglia, and test the effect of microglia-specific IL-6R knockout on AHN and cognitive function *in vivo*.

## Results

### IL-6 is expressed by multiple cell types in the SGZ, including NPCs, but expression of the IL-6R is only found in microglia.

**Figure 1:**
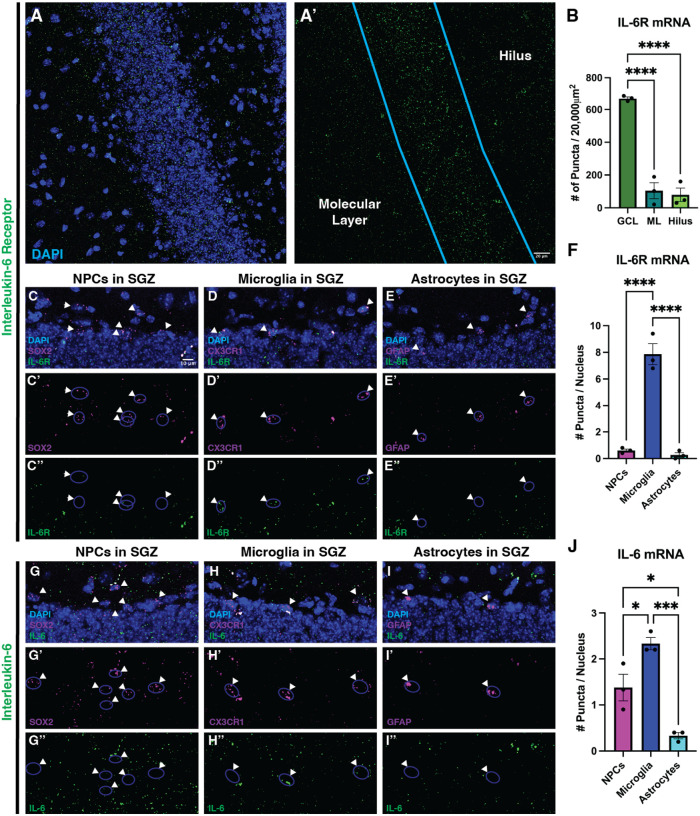
Microglia and NPCs express IL-6 but only microglia express IL-6R in the SGZ. RNAscope was used to label IL-6R mRNA in the DG, revealing that IL-6R expression is highly enriched in the GCL (A-B). Co-labeling with biomarkers of NPCs (C-C”), microglia (D-D”), and astrocytes (E-E”) revealed that IL-6R is highly enriched in microglia and not NPCs or astrocytes. Co-labeling of IL-6 mRNA with biomarkers of NPCs (G-G”), microglia (H-H”), and astrocytes (I-I”) revealed that IL-6 is expressed by microglia and NPCs but not astrocytes (J) *=P<.05, **=P<.01, ***=P<.001, ****=P<.0001. Scale bars are shared between A-A’ and C-I”.

Conflicting evidence concerning the expression of IL-6 and IL-6R in certain cell-types in the hippocampus necessitates a more focused investigation into the cell type-specific expression of these genes (24–25). Thus, we first sought to clarify the sources and expression levels of IL-6 and IL-6R within the adult DG. We utilized the RNAscope assay to label IL-6 and IL-6R mRNA. We quantified the number of IL-6 and IL-6R puncta within the molecular layer (ML), granule cell layer (GCL), and hilus of the DG (Figure 1A–A’; Supp. Figure 1A-A’), finding that IL-6 is expressed in each subregion of the DG, although there was a higher level of expression in the GCL than the hilus *[GCL: 2317 +/−152, ML: 2047 +/−205, Hilus: 1609 +/−324; F=5.3, P=.04.]* (Supp. Figure 1B). In contrast, IL-6R expression was most enriched in the GCL, suggesting that mature granule neurons express IL-6R *[GCL: 671.8 +/−11.0, ML: 104.2 +/−48.58, Hilus: 79.19 +/−40.8; F=81.14, P<.0001.]* (Figure 1B).

Next, we utilized Sox2, Cx3cr1, and GFAP as markers of NPCs, microglia, and astrocytes, respectively, to assess IL-6 and IL-6R expression across several of the most common cell-types in the SGZ. We quantified the number of IL-6R puncta colocalized with or in the immediate proximity of nuclei of Cx3cr1-, GFAP-, or Sox2-expressing cells (Figure 1C–E”), finding rare expression in astrocytes or NPCs, but consistently high expression in microglia *[Microglia: 7.86 +/−0.78, Astrocytes: 0.26 +/−0.17, NPCs: 0.60 +/−0.11; F=83.56, P<.0001.]* (Figure 1F). Quantification of IL-6 puncta (Figure 1G–I”) revealed that IL-6 was rarely expressed by astrocytes, commonly expressed by microglia, and variably expressed in neural progenitors from moderate levels of expression to no detectable levels *[Microglia: 2.33 +/−0.13, Astrocytes: 0.33 +/−0.06, NPCs: 1.37 +/−0.28; F=28.31, P=.0009.]* (Figure 1J). Thus, IL-6R is expressed primarily by microglia in the SGZ, while IL-6 is moderately expressed by microglia and NPCs and expressed at low levels in astrocytes.

### IL-6 classic- and *trans*-signaling induce nearly identical responses in microglia.

IL-6 has long been considered a pro-inflammatory cytokine, but recent evidence suggests that IL-6 classic- and *trans*- signaling have opposing effects on the activation state of microglia (26). Thus, we sought to better characterize the effect of different components of the IL-6 signaling cascade on microglia. We first tested the effect of treatment with recombinant IL-6 (rIL-6), recombinant IL-6R (rIL-6R), and Hyper-IL-6 (H-IL-6) on the adult-derived, immortalized murine microglia cell line (IMG). rIL-6R is the soluble fragment of the IL-6R, while H-IL-6 is a designer fusion protein of IL-6 and the soluble fragment of IL-6R which leads to strong IL-6 *trans* activation. Together, treatment of microglia with rIL-6, rIL-6R, and H-IL-6 recapitulate IL-6 classic stimulation, an IL-6R shedding event, and IL-6 *trans* stimulation, respectively.

**Figure 2:**
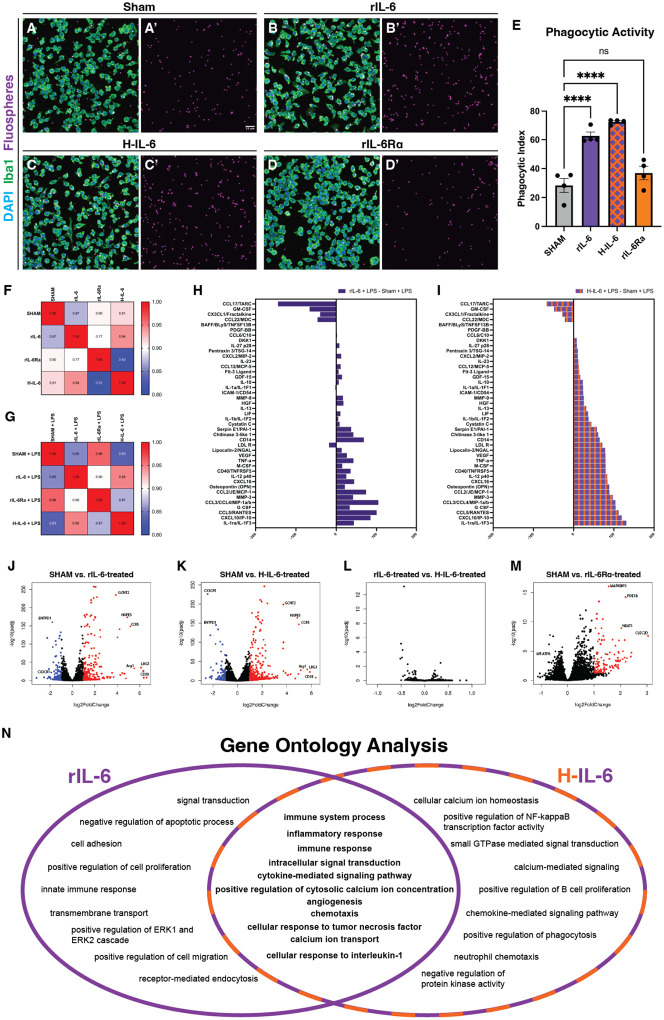
IL-6 classic and *trans*signaling yield a nearly identical pro-phagocytic and inflammatory response in microglia. IMG were treated with Fluospheres and either sham (A-A’), rIL-6 (B-B’), rIL-6R (C-C’), or H-IL-6 (D-D’) and incubated for 3 hours. The phagocytic index for microglia in each group was calculated and revealed that rIL-6 and H-IL-6 promote phagocytosis (E). IMG were then treated with sham, rIL-6, rIL-6R, or H-IL-6 or co-treated with each of these treatments plus LPS (F-G), again revealing a moderate but highly correlated response to rIL-6 and H-IL-6 (H-I). RNA sequencing confirmed that IMG treated with rIL-6 (J) and H-IL-6 (K) showed significant transcriptional changes, but no significant DEGs were detected between these two groups (L). rIL-6R yielded a distinct, unique transcriptional profile (M). GO analysis revealed the shared and unique pathways activated by rIL-6 or H-IL-6 (N). *=P<.05, **=P<.01, ***=P<.001, ****=P<.0001. Scale bars are shared between all images.

We first tested whether any of these treatments would alter the phagocytic activity of microglia by pretreating IMG *in vitro* with rIL-6, rIL-6R, H-IL-6, or a vehicle-control sham treatment. After 24 hours, polystyrene microspheres with a yellow-green fluorescent label and 1.0 μm diameter were added to each well and incubated for 3 hours. The cells were then fixed, washed with a 50% trypan blue solution to quench fluorescent signal from beads that were not phagocytosed, microglia were visualized with immunolabeling for iba1, and confocal images were collected (Figure 2A–D’). The phagocytic index for the microglia from each group was calculated, revealing that treatment with rIL-6 and H-IL-6 increased the phagocytic index of microglia, while rIL-6R had no effect *[Sham: 28.37 +/−4.88, rIL-6: 62.62 +/−2.7, rIL-6R : 36.93 +/−4.62, H-IL-6: 72.51 +/−0.87; F=32.65, P<.0001.]* (Figure 2E).

Next, we sought to determine the effect of these treatments on the microglia secretome. We utilized the Proteome Profiler Mouse XL Cytokine Array to analyze the relative levels of murine cytokines, chemokines, and growth factors in cell culture supernatants from IMG treated for 24 hours with rIL-6, rIL-6R, or H-IL-6 (Supp. Figure 2A). Each treatment yielded a distinct secretory profile, but the effects of rIL-6 treatment and H-IL-6 treatment yield a very high correlation coefficient (Figure 2F). IMG treated with rIL-6 or H-IL-6 were characterized by downregulation of CCL22 and CXCL2 and upregulation of CCL12 (Supp. Figure 2B-C). In contrast, rIL-6R treatment led to upregulation of IL-12 p40 and downregulation of CCL12 (Supp. Figure 2D). Next, we tested the effect of each treatment on the secretory profile of microglia activated by lipopolysaccharide (LPS) (Figure 2G, Supp. Figure 2A). Once again, the effects of rIL-6 and H-IL-6 were highly correlated, while rIL-6R yielded a secretory profile that only modestly differed from IMG receiving only LPS treatment (Figure 2G–I, Supp. Figure 2B-E). Compared to treatment with sham and LPS, IMG co-treatment with LPS and rIL-6 or LPS and H-IL-6 led to downregulation of CCL17, GM-CSF, CX3CL1, and CCL22 and upregulation of CCL2, CCL3, CCL5, CXCL10, G-CSF, and IL-1Ra, among others (Figure 2H–I).

Finally, we again treated IMG for 24 hours with rIL-6, rIL-6R, or H-IL-6 before collecting cell pellets and performing bulk RNA sequencing to assess transcriptional changes. Principal component analysis of bulk RNA sequencing revealed that each treatment induced a distinct transcriptional profile in microglia relative to sham (Supp. Figure 2 F-H). Volcano plots reveal that rIL-6 and H-IL-6 had similar effects on transcription, characterized by upregulation of CCR5, ARG1, and CD38 and downregulation of CX3CR1 and ENTPD1 (Figure 2J–K). When comparing rIL-6- and H-IL-6-treated IMG, there were no differentially expressed genes that reached statistical significance (Figure 2L). Treatment with rIL-6R yielded a unique transcriptional response characterized by upregulation of NEAT1 and CLEC2D (Figure 2M).

Gene ontology (GO) analysis revealed significant overlap between the activated canonical pathways in rIL-6- and H-IL-6-treated microglia. The top GO terms that were activated in both groups were related to immune regulation, cytokine-mediated signal transduction, calcium homeostasis, and chemotaxis (Figure 2N). However, some GO terms were only found in one group, such as “positive regulation of cell proliferation” and “negative regulation of apoptotic process” only being detected after rIL-6 treatment. Finally, we assessed whether genes associated with the GO terms that were shared between rIL-6- and H-IL-6-treated microglia shared the same directional regulation and magnitude of change. We found that the expression of markers of the immune response and a homeostatic cell state was nearly identical in both groups [Supp. Table 1] (Supp. Figure 2I-K). In sum, our results points to IL-6 classic- and *trans*-signaling having nearly identical effects on microglia, characterized by inflammatory regulation, promotion of phagocytosis, and induction of a neuroprotective phenotype.

### IL-6 signaling regulates the proliferation and neuronal differentiation of NSCs.

Findings from embryonic forebrain rodent NSCs suggest that IL-6 classic- and *trans*-signaling regulate their glial and neuronal differentiation (15,18–19). However, evidence from adult rodents *in vivo* suggests that IL-6 *trans*-signaling only promotes neuronal survival (25). Thus, the effect of IL-6 classic- or *trans*-signaling on the state or function of adult-derived hippocampal NSCs remains unclear.

**Figure 3:**
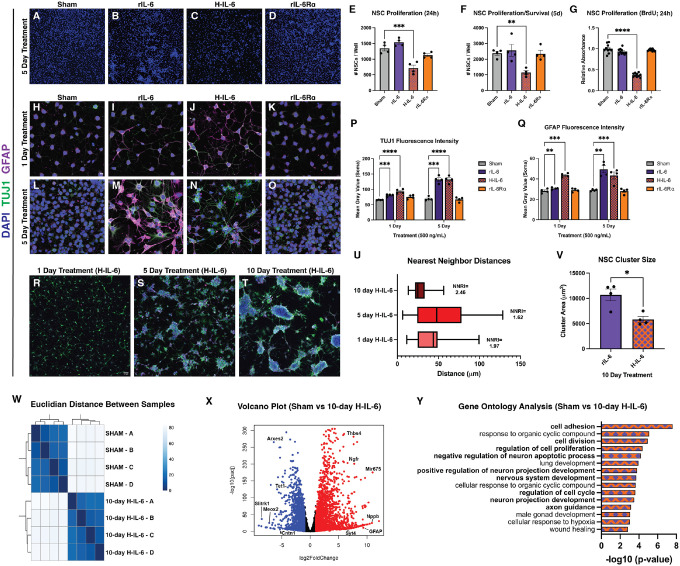
IL-6 *trans* signaling in AH-NSCs arrests proliferation and induces neuronal differentiation, spatial reorganization, and activation of molecular pathways associated with neuronal maturation. AH-NSCs were treated with sham (A), rIL-6 (B), H-IL-6 (C), or rIL-6R (D) and proliferation was measured via cell counting (E-F) or the BrdU assay (G), revealing that only H-IL-6 arrests AH-NSC proliferation. Performing these same treatments for 1 day (H-K) or 5 days (L-O) and measuring fluorescence intensity for TUJ1 (P) or GFAP (Q) revealed increased immunoreactivity for each protein following rIL-6 and H-IL-6 treatment. AH-NSCs treated for 1 day (R), 5 days (S) or 10 days (T) revealed that H-IL-6 induced spatial reorganization into clusters (U). AH-NSCs treated with rIL-6 also reorganized into clusters, but also maintained their proliferative state (V). RNA sequencing of naïve AH-NSCs and AH-NSCs treated for 10 days with H-IL-6 revealed a significant, consistent response (W) characterized by an increase in expression of neuronal genes and decrease in expression of stem cell genes (X). GO analysis revealed that many activated molecular pathways in H-IL-6-treated AH-NSCs are related to cell-cycle regulation and neuronal maturation (Y) *=P<.05, **=P<.01, ***=P<.001, ****=P<.0001. Scale bars are shared between A-D, H-O, and R-T.

Therefore, we treated rodent adult hippocampal neural stem cells (AH-NSCs) with sham, rIL-6, H-IL-6, or rIL-6R for 1 day and 5 days to test the effect of treatments on proliferation and a mix of proliferation and survival, respectively (Figure 3A–D; Supp. Figure 3A-D). Analyses of cell density revealed that only H-IL-6 caused a significant reduction in the number of cells per well at 1 day and 5 days compared to sham-treated cells *[Sham: 1348 +/−81.78, rIL-6: 1540 +/−61.56, H-IL-6: 720 +/−94.23, rIL-6R : 1130 +/−45.30; F=23.16, P<.0001.] [Sham: 2381 +/−132.7, rIL-6: 2564 +/−374.6, H-IL-6: 1146 +/−126.7, rIL-6R : 2349 +/−212.6; F=7.70, P=.0039.]* (Figure 3E–F). The 1-day BrdU intake assay also showed that only H-IL-6 arrested the proliferation of AH-NSCs *[Sham: 1.00 +/−0.03, rIL-6: 0.93 +/−0.02, H-IL-6: 0.37 +/−0.01, rIL-6R : 0.97 +/−0.008; F=195.2, P<.0001.]* (Figure 3G).

We next determined whether rIL-6, H-IL-6, or rIL-6R affects the differentiation of AH-NSCs. After 1 day or 5 days, fixed cells were immunolabeled with antibodies that target mature neurons (βIII-tubulin, TUJ1) or both astrocytes and a subset of neural precursors (GFAP) (Figure 3H–O). The fluorescence intensity of TUJ1 and GFAP in the soma of 10 randomly sampled cells per well were recorded. In the sham and rIL-6R groups, the AH-NSCs continued to proliferate until they reached 100% confluence (Figure 3H–O). Receiving rIL-6 or H-IL-6 treatment increased TUJ1 and GFAP fluorescence intensity [Supp. Table 2] (Figure 3P–Q). We repeated these experiments, this time using titrations of either rIL-6 alone or co-treatment of rIL-6 and rIL-6R to test whether the addition of rIL-6R, and presumably the formation of the rIL-6/rIL-6R protein complex that activates IL-6 *trans*-signaling, would alter the potency of these treatments (Supp. Figure 3F). We found that rIL-6 and rIL-6R treatment was able to cause a significant increase in TUJ1 fluorescence intensity compared to rIL-6-only treatment after 24 hours at a concentration of only 15.625 ng/ml, indicating that rIL-6R and rIL-6 co-treatment is more potent than rIL-6 alone in this context [Supp. Table 3] (Supp. Figure 3G). Further, we found that co-treatment of rIL-6 and rIL-6R was able to cause a significant increase in GFAP fluorescence intensity compared to rIL-6-only treatment after 24 hours at a minimal concentration of 62.5 ng/ml [Supp. Table 4] (Supp. Figure 3H).

Next, we tested whether H-IL-6 led to changes in spatial organization of AH-NSCs. To test this, we performed the nearest neighbor analysis on AH-NSCs treated for 1, 5, or 10 days (22) (Figure 3R–T). The treatment led to spatial reorganization into clusters, as evidenced by the increase in the nearest neighbor regularity index (NNRI) from 5 days to 10 days *[H-IL-6 1 Day: 42.72 +/−4.84, H-IL-6 5 Day: 53.23 +/−7.323, H-IL-6 10 Day: 28.83 +/−2.61; F=5.35, P<.0074.]* (Figure 3U). Because both rIL-6-treated and H-IL-6-treated AH-NSCs differentiated, while only H-IL-6-treated AH-NSCs stopped proliferating (Figure 3E–G,P–Q), we tested whether the clusters of AH-NSCs were of different sizes in each group. As expected, we found that clusters of AH-NSCs treated with rIL-6 were approximately double the size of those from cells treated with H-IL-6 *[rIL-6: 10695 +/−1176, H-IL-6: 5824 +/−589.5; P=.01.]* (Figure 3V, Supp. Figure 3I).

Finally, we performed bulk RNA sequencing on AH-NSCs treated for 10 days with sham or H-IL-6 to assess transcriptional changes in NSCs induced by IL-6-*trans* signaling. Comparison of the Euclidian distance between samples revealed that H-IL-6 treatment led to significant transcriptional changes that were highly consistent between replicates (Figure 3W). Volcano plots for the top DEGs between the two groups showed that H-IL-6 led to upregulation of key markers of neuronal differentiation including THBS3, NGFR, MIR675, NPPB, GFAP, and SYT4, as well as downregulation of multiple genes associated with the neural stem cell state, including ARXES2, TET1, SLITRK1, MEOX2, and CNTN1 (Figure 3X). Finally, GO analysis revealed that H-IL-6 treatment led to the activation of GO pathways primarily associated with cell cycle regulation, neuronal development, axon guidance, cell migration and adhesion, and negative regulation of apoptotic processes (Figure 3Y). These findings indicate that IL-6 *trans*-signaling arrests proliferation and induces neuronal differentiation and maturation in AH-NSCs.

### The efferocytosis of apoptotic NSCs leads to IL-6R shedding by microglia.

The apoptosis of NSCs is a common occurrence in the adult DG, and the removal of these cells is a critical role of ramified hippocampal microglia that maintains homeostasis in the SGZ (12). However, the spatiotemporal organization of microglia in response to NPC proliferation and apoptosis are not well described, nor are the location (relative to proliferating clusters) and identity of apoptotic cells in the SGZ. We therefore sought to clarify the organization of microglia, proliferating NPCs, and apoptotic NPCs in the SGZ of adult mice.

**Figure 4:**
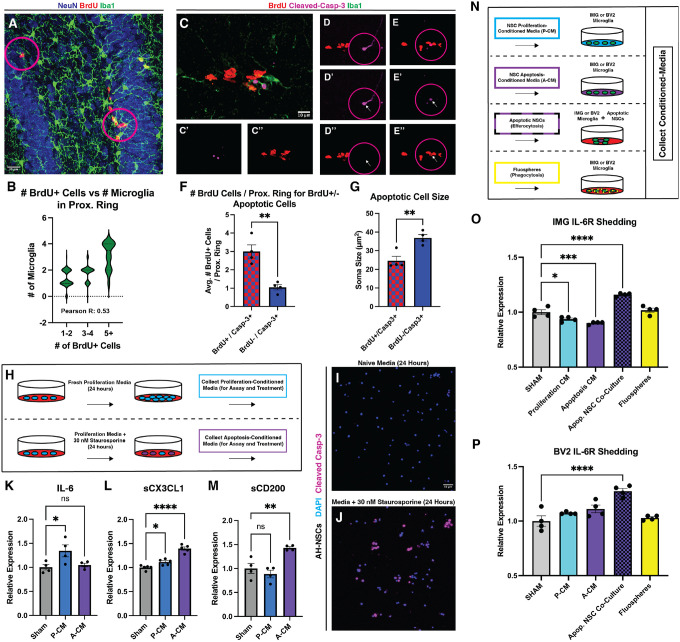
Microglia aggregate at large clusters of proliferating NPCs, efferocytose apoptotic NPCs within these clusters, and shed the IL-6R during efferocytosis. Young adult wild-type mice received BrdU injections and were sacrificed after 24 hours. Assessments of the spatial organization of microglia and newborn cells in the SGZ revealed a correlation between microglia number and BrdU cell number (A-B). BrdU+ apoptotic cells were often found being engulfed by ramified microglia within clusters of viable NPCs, while BrdU- apoptotic cells were larger in size and were efferocytosed by microglia away from clusters of proliferating NPCs (C-G). We probed the conditioned media of proliferating or apoptotic AH-NSCs (H-J) and found that IL-6 was enriched in P-CM (K), sCD200 was enriched in A-CM (M), and sCx3cl1 was enriched in both conditions (L). We then tested whether P-CM, A-CM, an efferocytosis challenge, or a synthetic phagocytosis challenge would induce IL-6R shedding in microglia (N), finding that, of these, only efferocytosis causes IL-6R shedding by microglia (O-P) *=P<.05, **=P<.01, ***=P<.001, ****=P<.0001. Scale bars are shared between C-E” and I-J.

We assayed the aggregation of microglia in the SGZ of adult wild-type mice at proliferating or apoptotic NPCs by performing BrdU injections and sacrificing the mice after 24 hours. Immunohistochemistry was used to label BrdU, iba1, and cleaved-caspase-3 to label cells that divided within the last 24 hours, microglia, and apoptotic cells, respectively. We overlayed a circle (which we have termed a ‘proximity ring’) with a 2,000 μm^2^ area on each image, centered on each BrdU+ cell in the SGZ. We then counted the number of microglia, BrdU+ cells, and cleaved-caspase-3+ (Casp-3+) cells within each proximity ring (Figure 4A). We found that the number of microglia and the number of BrdU+ cells in each proximity ring is highly correlated *[1–2 BrdU Cells: 1.37 +/−0.08, 3–4 BrdU Cells: 1.77 +/−0.07, 5+ BrdU Cells: 3.125 +/−0.25; R=0.53, P<.0001.]* (Figure 4B). Unsurprisingly, Casp-3+ cells were often found in the process of being engulfed by ramified microglia (Figure 4C–C”). Additionally, we found that newborn apoptotic cells (BrdU+/Casp-3+) were more often found near viable newborn cells (BrdU+/ Casp-3-) than were cells that became apoptotic sometime after the first day of life (BrdU-/Casp-3+) *[BrdU+/Casp-3+: 3.00 +/−0.36, BrdU-/Casp-3+: 1.04 +/−0.14; P=.0024.]* (Figure 4C–F). We also found that these BrdU- apoptotic cells tended to be of a larger size than BrdU+ apoptotic cells and often included a labeled extension projecting toward the molecular layer of the DG, further pointing to these cells that become apoptotic away from clusters of proliferating cells being more mature than their BrdU+ counterparts *[BrdU+/Casp-3+: 24.62 +/−2.31, BrdU-/Casp-3+: 36.91 +/−1.78; P=.0056.]* (Figure 4D–D”, G).

Having determined that newborn cells in the SGZ often become apoptotic and are removed by microglia while near numerous viable NPCs, we tested whether microglial efferocytosis leads to IL-6R shedding. To test this, we treated AH-NSCs *in vitro* with either 30 nM staurosporine to induce apoptosis or a sham treatment for 24 hours (Figure 4H). Cleaved-caspase-3 immunoreactivity was only detected in staurosporine-treated cells (Figure 4I–J). After 24 hours, the media from each well was collected and either stored for later analysis or pooled to use as a treatment. This pooled conditioned media (CM) from either proliferating NSCs (P-CM) or apoptotic NSCs (A-CM), was concentrated 5x using Pierce protein concentrators and then resuspended to 1x in NSC media.

After verifying that P-CM and A-CM do not induce apoptosis in IMG (Supp. Figure 4), we probed the CM from each condition for IL-6 by ELISA, finding that only P-CM showed increased IL-6 levels compared to unconditioned media *[Sham: 1.00 +/−0.05, AH-NSC P-CM: 1.34 +/−0.12, AH-NSC A-CM: 1.047 +/−0.03; F=5.27, P=.03.]* (Figure 4K). We next tested P-CM and A-CM for the soluble fragments of the transmembrane proteins CX3CL1 and CD200, which are expressed on neurons and known to activate microglia and act as potent chemokines when shed from the cell surface. We found a minor increase in sCX3CL1 in P-CM, but a much more robust increase in both sCX3CL1 and sCD200 in A-CM *[Sham: 1.00 +/−0.02, AH-NSC P-CM: 1.11 +/−0.02, AH-NSC A-CM: 1.39 +/−0.03; F=50.02, P<.0001.] [Sham: 1.00 +/−0.21, AH-NSC P-CM: 0.88 +/−0.07, AH-NSC A-CM: 1.431 +/−0.02; F=13.73, P=.0018.]* (Figure 4L–M).

Next, IMG were then either co-plated 5:1 with apoptotic AH-NSCs or treated with P-CM, A-CM, or sham for 24 hours, after which the media was collected for analysis. Together, this experiment allows us to probe the response of microglia stimulated by the secretome of proliferating NSCs, microglia stimulated by the secretome of apoptotic NSCs, and microglia stimulated by apoptotic NSCs (induces efferocytosis by microglia). We also probed microglia treated with fluospheres to test whether any phagocytic challenge induced IL-6R shedding (Figure 4N). Assessment of IL-6R protein levels in the conditioned media from each group revealed that only co-culture of microglia and apoptotic NSCs led to an increase in IL-6R shedding *[Sham: 1.00 +/−0.22, AH-NSC P-CM: 0.93 +/−0.01, AH-NSC A-CM: 0.93 +/−0.00, Apoptotic AH-NSC Co-Culture: 1.16 +/−0.00, Fluospheres: 1.08 +/−.01; F= 45.80, P<.0001.]* (Figure 4O). This experiment was repeated in another common microglia cell line (BV2), which yielded the same result *[Sham: 1.00 +/−0.04, AH-NSC P-CM: 1.072 +/−0.01, AH-NSC A-CM: 1.11 +/−0.07, Apoptotic AH-NSC Co-Culture: 1.27 +/−0.05, Fluospheres: 1.02 +/−.02; F= 13.30, P<.0001.]* (Figure 4P). These findings reveal that apoptotic NPCs in the SGZ are most often found within clusters of viable NPCs, that microglia aggregate at these large clusters of NPCs to remove the apoptotic cell, and that microglia shed the IL-6R during efferocytosis of apoptotic NPCs.

### IL-6R knockout in microglia causes impairments in adult neurogenesis and cognitive behavioral deficits in contextual learning and memory.

**Figure 5:**
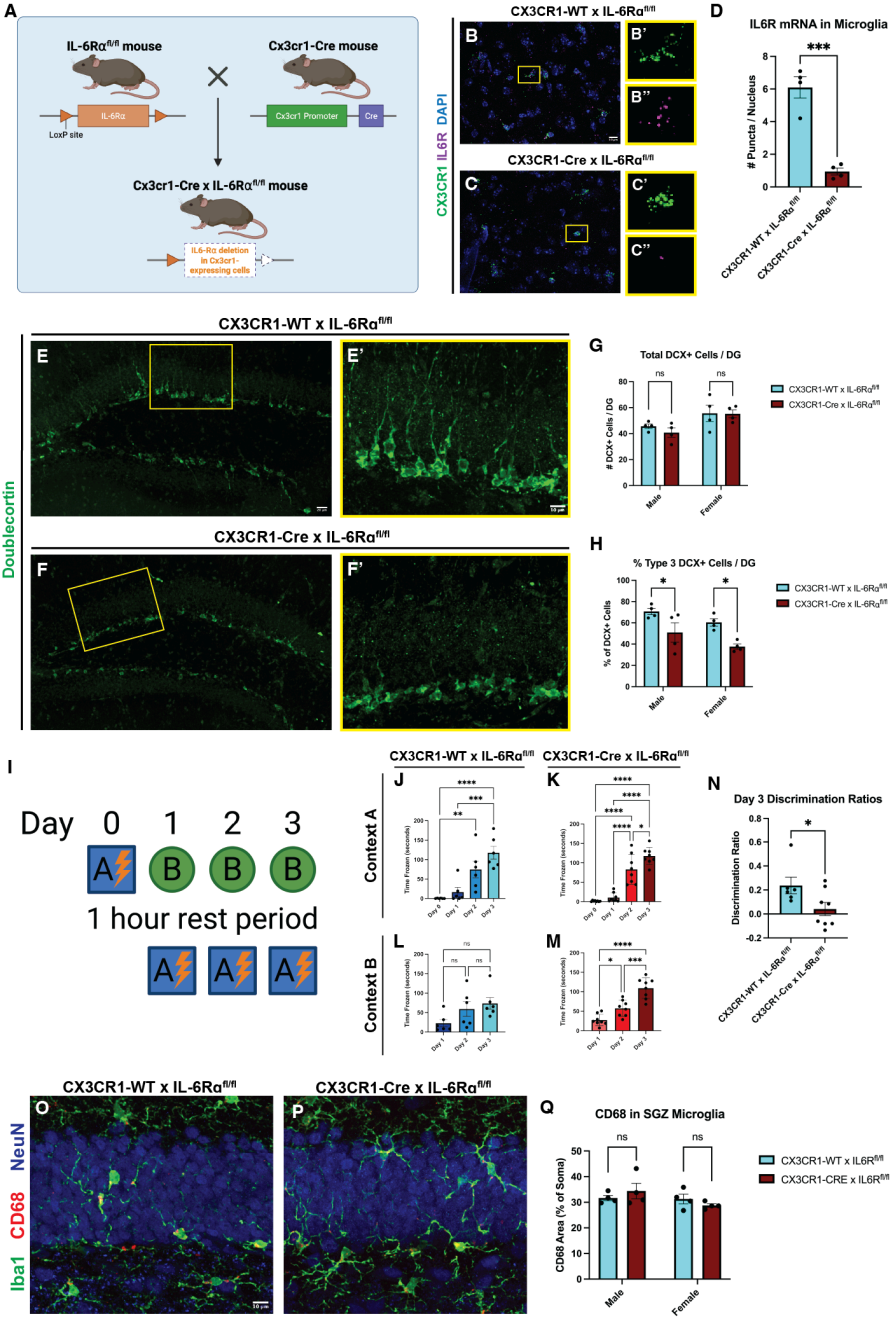
Loss of IL-6R expression in microglia causes cellular deficits in neurogenesis that result in impaired learning and memory. We generated mice lacking IL-6R exclusively in microglia using the Cre-Lox system (A). Loss of IL-6R expression in double-transgenic mice was confirmed by RNAscope (B-D). Immunolabeling of DCX revealed that the total number of DCX+ cells is unchanged (E-G) but that the percentage of DCX+ cells that were of Type 3 was significantly reduced (H). We performed the contextual fear conditioning assay on additional cohorts of male and female mice (I) finding that loss of microglial IL-6R causes deficits in contextual fear memory (J-N). CD68 levels in microglia were quantified by measuring area of CD68 signal in microglia soma and calculating the percentage of microglia soma occupied by CD68 signal (O-Q) *=P<.05, **=P<.01, ***=P<.001, ****=P<.0001. Scale bars are shared between E+F, E’+F’ and O+P.

Despite evidence implicating both microglia and IL-6 signaling as regulators of neurogenesis, it remains unknown whether microglial IL-6 signaling is a dominant mechanism in cognitive and behavioral functions that rely on AHN (27–28). Our other findings herein suggest that IL-6R expression in microglia could be a critical factor in the regulation of AHN. Thus, we crossed Cx3cr1-Cre mice with IL-6R ^fl/fl^ mice to generate a new mouse line in which microglia do not express IL-6R (Figure 5A; Supp. Figure 5A-B”). This mouse line impairs the ability of microglia to respond to IL-6, thus impairing microglial IL-6 classic-signaling. It also removes the ability of microglia to shed the IL-6R, which blocks microglia from inducing IL-6 *trans* signaling.

First, we validated the IL-6R knockout using RNAscope to label mRNA for Cx3cr1 and IL-6R. Cx3cr1-Cre x IL-6R^fl/fl^ mice show a clear, consistent loss of IL-6R mRNA expression in microglia compared to Cx3cr1-WT x IL-6R^fl/fl^ *[Cx3cr1-WT;IL-6R* ^*fl/fl*^*: 6.10 +/−0.64, Cx3cr1-Cre;IL-6R* ^*fl/fl*^*: 0.95 +/−0.21; P=.0003.]* (Figure 5B–D; Supp. Figure 5A-D”). We also validated that IL-6R-expression in mature dentate granule neurons was unchanged in transgenic mice, indicating that IL-6R knockout is restricted to microglia as intended (Supp. Figure 5A-B). We performed immunohistochemistry to label iba1 and assayed the density of microglia in the DG and SGZ, finding no change between groups *[CX3CR1-WT x IL-6R* ^*fl/fl*^*: 165.3 +/−9.90, CX3CR1-Cre x IL-6R* ^*fl/fl*^*: 158.8 +/−6.21; P=.59.] [CX3CR1-WT x IL-6R* ^*fl/fl*^*: 38.75 +/−2.83, CX3CR1-Cre x IL-6R* ^*fl/fl*^*: 34.75 +/−3.35; P=.39.]* (Supp. Figure 5E-H). We also found no changes in tiling regularity or in the percentage of microglia with a ramified morphology *[CX3CR1-WT x IL-6R* ^*fl/fl*^*: 3.11 +/−0.08, CX3CR1-Cre x IL-6R* ^*fl/fl*^*: 3.43 +/−0.30; P=.34.] [CX3CR1-WT x IL-6R fl/fl: 93.39 +/−0.93, CX3CR1-Cre x IL-6R fl/fl:94.49 +/−0.47; P=.33.]* (Supp. Figure 5E-F,I-J).

Next, we performed immunostaining for doublecortin (DCX) to test whether IL-6R knockout exclusively in microglia was sufficient to disrupt neurogenesis *in vivo*. We found that the number of DCX+ cells in the SGZ was unchanged in male and female Cx3cr1-Cre x IL-6R^fl/fl^ mice compared to controls *[Male Cx3cr1-WT;IL-6R* ^*fl/fl*^*: 45.63 +/−1.74, Male Cx3cr1-Cre;IL-6R* ^*fl/fl*^*: 40.75 +/−3.59, Female Cx3cr1-WT;IL-6R* ^*fl/fl*^*: 55.63 +/−6.29, Female Cx3cr1-Cre;IL-6R* ^*fl/fl*^*: 55.25 +/−2.99; Male P=0.64, Female P=0.99.]* (Figure 5E–G). Finally, we counted the number of type-1, type-2, or type-3 DCX+ immature neurons as described previously (23), finding a significant reduction in the number of type 3 DCX+ cells in both male and female Cx3cr1-Cre x IL-6R^fl/fl^ mice, indicating impaired maturation of NPCs *[Male Cx3cr1-WT;IL-6R* ^*fl/fl*^*: 70.68 +/−3.0, Male Cx3cr1-Cre;IL-6R* ^*fl/fl*^*: 50.92 +/−9.0, Female Cx3cr1-WT;IL-6R* ^*fl/fl*^*: 60.45 +/−3.45, Female Cx3cr1-Cre;IL-6R* ^*fl/fl*^*: 37.59 +/−2.47; Male P=0.04, Female P=0.01.]* (Figure 5E–F’,H).

Next, we performed the contextual fear conditioning assay using male and female Cx3cr1-Cre x IL-6R^fl/fl^ mice and age- and sex-matched Cx3cr1-WT x IL-6R^fl/fl^ controls to determine if depletion of microglial IL-6R is sufficient to cause deficits in hippocampus-dependent learning and memory (Figure 5I). Both Cx3cr1-WT x IL-6R^fl/fl^ *[Day 0: 0.33 +/−0.21, Day 1: 16.80 +/−11.58, Day 2: 74.80 +/−21.41, Day 3: 117.7 +/−16.45; F=13.50, P<.0001.]* (Figure 5J) and Cx3cr1-Cre x IL-6R^fl/fl^ *[Day 0: 1.37 +/−0.53, Day 1: 10.83 +/−4.194, Day 2: 83.31+/−13.52, Day 3: 117.6 +/−7.82; F= 48.55, P<.0001.]* (Figure 5K) mice acquired a learned fear response to the shock context. When placed in the same test-chamber but with different context clues, Cx3cr1-WT x IL-6R^fl/fl^ mice were able to distinguish a similar context from the shock context, indicated by no significant increase in freezing behavior in the safe context *[Day 1: 22.72 +/−9.52, Day 2: 59.48 +/−19.12, Day 3: 73.75 +/−14.99; F=3.05, P=.07.]* (Figure 5L). However, mice lacking IL-6R expression in microglia were unable to distinguish between the shock context and the safe context, as indicated by increased freezing behavior in the safe context for Cx3cr1-Cre x IL-6R^fl/fl^ mice *[Day 1: 27.53 +/−5.14, Day 2: 57.61 +/−7.32, Day 3: 109.3 +/−9.34; F=30.64, P<.0001.]* (Figure 5M). We calculated the discrimination ratios for the final day of trials, finding that contextual fear discrimination is significantly impaired in male and female mice lacking IL-6R in microglia *[Cx3cr1-WT;IL-6R* ^*fl/fl*^*: 0.23 +/−0.06, Cx3cr1-Cre;IL-6R* ^*fl/fl*^*: 0.04 +/−0.05; P=.04.]* (Figure 5N).

Finally, we performed immunostaining for the microglia marker iba1, the mature neuron marker NeuN, and the phagocytic marker CD68 to determine whether SGZ microglia lacking IL-6R showed alterations to their phagocytic activity (Figure 5O–P). We measured the area of the microglia soma and the total area of CD68 signal within microglia soma in the SGZ and calculated the average percentage of the soma area taken up by CD68. We compared these values between male and female Cx3cr1-WT x IL-6R^fl/fl^ and Cx3cr1-Cre x IL-6R^fl/fl^ mice, finding that there was no difference in the level of CD68 within SGZ microglia across genotypes *[Male Cx3cr1-WT;IL-6R* ^*fl/fl*^*: 31.67 +/−1.03, Male Cx3cr1-Cre;IL-6R* ^*fl/fl*^*: 34.42 +/−3.03, Female Cx3cr1-WT;IL-6R* ^*fl/fl*^*: 31.30 +/−1.88, Female Cx3cr1-Cre;IL-6R* ^*fl/fl*^*: 28.75 +/−0.67; Male P=0.64, Female P=0.71.]* (Figure 5Q). These findings indicate that loss of microglial IL-6R is sufficient to impair NPC maturation and confer cognitive deficits in neurogenesis-dependent learning and memory tasks, but not to alter microglial phagocytic activity or the total number of DCX+ cells.

## Discussion

Our findings reveal a previously unrecognized mechanism by which microglia regulate AHN through dynamic interactions with apoptotic NPCs, and identify IL-6 *trans*-signaling as a critical modulator of phagocytosis, inflammatory tone, and NPC fate specification. We demonstrate that: (1) clusters of proliferating NPCs secrete IL-6, and a subset of these cells inevitably undergo apoptosis; (2) microglia are recruited to these clusters to clear apoptotic NPCs, during which they shed IL-6R, which binds IL-6 secreted by viable NPCs to form the IL-6 *trans*-signaling complex (IL-6/IL-6R); (3) this complex facilitates the phagocytic removal of apoptotic NPCs by microglia and promotes a shift toward a neuroprotective state; and (4) concurrently, the IL-6 *trans* protein complex inhibits NPC proliferation while enhancing neuronal differentiation and maturation of surviving NPCs.

We herein perform myriad *in vitro* assays in microglia to assess the effect of IL-6 signaling molecules on mRNA transcription, cytokine secretion, cell state, and function. IL-6 has been extensively studied in both the central and peripheral nervous system, with the consensus of the literature finding IL-6 to be pro-inflammatory and to induce chemotaxis in immune cells (23,26). However, there is also evidence that chronic IL-6 classic-signaling yields an anti-inflammatory response (34). The detection of increased levels of IL-6 during the onset of neurodegenerative diseases like AD (35), which is in part characterized by increased neuroinflammation, strengthens the link between IL-6 and inflammation regulation in disease contexts. In contrast, IL-6 *trans*-signaling has received much less attention, and its role in the brain is comparatively unclear, although *trans*-signaling is most often linked to pro-inflammatory outcomes (34). The effect of the soluble fragment of IL-6R on microglia has gone almost entirely uninvestigated, all of which prompted us to systematically assess the effects of IL-6 classic-signaling, IL-6 *trans*-signaling, and the soluble fragment of IL-6R on microglia. Our finding that IL-6 classic- and *trans*-signaling yield nearly identical outcomes in microglia aligns with expectations in some ways, given that both modalities converge on gp130 activation (23). However, given the evidence linking IL-6 classic-signaling to anti-inflammatory outcomes and *trans*-signaling to pro-inflammatory outcomes, further investigation is required to fully understand how these signaling modalities regulate microglia *in vivo*, since the contributions of other cell types and the overall tissue microenvironment may guide the response of microglia to classic- or *trans*-signaling activation. Our finding that IL-6 *trans*-signaling activation increased SOCS3 expression, which is well described as a downstream target of IL-6 classic-signaling, draws additional scrutiny onto the belief that IL-6 *trans*-signaling is purely pro-inflammatory. This is also the first time, to our knowledge, that any effect of IL-6Ra on microglia has been measured. The unique response of microglia to this molecule requires further investigation to fully understand its effect and relevance *in vivo*.

We performed a similar systematic *in vitro* analysis of the effect of the primary components of IL-6 signaling in NSCs. Compared to microglia, there is a dearth of evidence for the effect of IL-6 signaling on NSCs, especially in the context of IL-6 *trans*-signaling and adult neurogenesis. However, the evidence that does exist, such as the finding from Islam et al. that *trans*-activating fusion protein H-IL-6 promotes the expression of differentiation markers, including markers of neurogenesis and gliogenesis in embryonic NSC-derived neurospheres, is largely consistent with our findings (15). In addition, our findings also reveal that while rodent NSCs do respond to IL-6, a very high concentration is required to yield a cellular response. Our finding that NSCs respond to much lower concentrations of IL-6 when co-treated with rIL-6Ra suggests that these cells express low levels of IL-6R and comparatively high levels of gp130.

Our Cx3cr1-Cre x IL-6R^fl/fl^ mouse model provides an opportunity to test which of our *in vitro* findings are dominant regulators of the associated cell functions *in vivo*. Our findings presented in Figure 2 link gp130 activation via H-IL-6 to molecular pathways associated with neurite outgrowth and guidance and cell-cycle regulation. In our microglial IL-6R knockout mouse model, we detected severe impairments in neurite development in DCX+ cells in the SGZ, but no changes in overall DCX+ cell number. This suggests that microglia are dominant regulators of NPC maturation and that IL-6R expression is critical to this regulation. In contrast, despite linking IL-6 *trans* activation to cell cycle regulation *in vitro*, we detected no change in overall NPC density *in vivo* in Cx3cr1-Cre x IL-6R^fl/fl^ mice. Similarly, we found that IL-6 classic-and *trans*-signaling promote phagocytosis by microglia, but loss of microglial IL-6R did not confer altered levels of CD68 *in vivo*. This finding that some, but not all, of our findings *in vitro* are recapitulated *in vivo* provides evidence as to which of these cell functions we have linked to IL-6 signaling likely have redundant or compensatory mechanisms *in vivo*. These findings also warrant an even more focused assessment of the effect of IL-6 signaling molecules on neural progenitors, as NSCs, neuroblasts, and immature granule cells all demonstrate unique signatures that may include differing responses to IL-6.

Links between IL-6 and the regulation of neurogenesis *in vivo* have slowly been uncovered over recent decades (15–18,20,27). Constitutive IL-6 knockout mice have been found to have impairments in neural progenitor proliferation, differentiation, maturation, and survival in several brain regions where neurogenesis occurs (27). Here, we find that some, but not all, of these impairments can be directly linked to microglia. Together, these studies point to IL-6 as being a regulator of neurogenesis across the neurogenic niches of the brain, meaning that our findings herein may also be relevant to neurogenesis outside the hippocampus. Although highly informative, these studies often lack a cell-type specific or mechanistic explanation for the effect of experimental IL-6 modulation on neurogenesis. Therefore, future work should focus on assessing the relevance of efferocytosis-linked microglial regulation in these other brain regions where IL-6 and/or microglia have been implicated as regulators of neurogenesis.

Evidence for IL-6 *trans*-signaling as a regulator of neurogenesis is sparse. Additionally, there is no consensus on whether *trans*-signaling is neuroprotective or neurotoxic. Campbell et al. found that *trans*-signaling is a dominant mechanism for the pathogenic actions of IL-6 in the brain (36). Meanwhile, Willis et al. show that IL-6 *trans*-signaling is critical to the recovery of normal neurogenesis following traumatic brain injury by promoting neuronal survival in the hippocampus (14). Together with our findings herein, these manuscripts highlight the need for a comprehensive analysis of the effect of IL-6 *trans*-signaling on neurogenesis in healthy, disease, and injury contexts.

Like IL-6, the number of links between microglia and neurogenesis has slowly grown over the past two decades (11–12,14,28,37). Sierra et al. demonstrated that efferocytosis is a critical function of microglia in maintaining homeostasis in the SGZ (12). Diaz-Aparicio et al. posited that the microglial phagocytic secretome was part of a negative feedback loop that contributed to the long-term maintenance of adult neurogenesis (11). Here, we present a molecular mechanism that supports these hypotheses and provides a molecular target for exogenous regulation of neurogenesis through microglia. Our findings herein also link IL-6 signaling in microglia to ARG1 upregulation. ARG1+ microglia express high levels of insulin-like growth factor 1 (IGF1), a known neurogenic factor secreted by microglia, and ARG1 knockdown in microglia impaired dendritic spine maturation in the hippocampus (38). These findings further strengthen the link between microglial IL-6 signaling, inflammation regulation, and neurite development.

Our results position microglia not merely as passive responders to cell death but as active regulators of lineage specification and progenitor cell maturation within the neurogenic niche. The IL-6 *trans* signaling axis appears to function as a temporally gated checkpoint that coordinates niche refinement—balancing expansion with maturation and synchronizing neuronal development with microglial activation and quiescence. However, in pathological contexts such as AD, chronic or excessive IL-6 *trans* signaling may impose sustained suppression of NPC proliferation and premature differentiation, ultimately depleting the progenitor pool and reducing net AHN. Collectively, our data identify IL-6 *trans* signaling, triggered by microglial crosstalk with apoptotic NSCs, as a pivotal molecular switch linking phagocytosis, inflammation resolution, and neurogenic output, and suggest that targeted modulation of this pathway may offer therapeutic potential for restoring AHN in aging and neurodegenerative diseases.

### Limitations

In this study, we show that only microglia express IL-6R, while microglia, NPCs, and astrocytes all express low levels of IL-6 in the SGZ. These findings were based on mRNA expression, and protein-level validation in each cell type are warranted to fully understand the expression patterns of these genes in the SGZ. Our study used immortalized microglial lines and conditional knockout mice, which may not fully reflect microglial diversity or functional states influencing IL-6 signaling and neurogenesis. While our IL6R shedding data from IMG were reproduced in BV2 microglia, future work should investigate which of our findings translate to primary microglia. The IL-6R knockout may also trigger compensatory changes in other cytokine pathways, complicating causal interpretation. Additionally, we only assayed NSCs and not NPCs or neuroblasts *in vitro*. Further experimentation is needed to fully understand the effect of IL-6 classic- and *trans*-signaling on these cells. Finally, our Cx3cr1-Cre x IL-6Ra^fl/fl^ mice effect microglia throughout the brain, not just in the hippocampus, as well as the small subset of Cx3cr1-expressing cells in the brain that are not microglia, such as border-associated macrophages, which are largely excluded from the brain parenchyma. While the constitutive Cx3Cr1-Cre mouse line provides a robust method for knocking out IL-6R from microglia, it does so throughout the animals’ lives. Future work should assess the effect of IL-6R knockout on neurodevelopment and neurogenesis during early life. While technically challenging, future experiments should assess the effect of blocking IL-6 classic signaling or IL-6 *trans* signaling in isolation to better elucidate the contribution of each signaling pathway to adult neurogenesis *in vivo*.

## Materials and Methods

### Experimental Models:

#### Mice

Young adult (4 months) C57BL/6J (RRID: IMSR_JAX:000664), Cx3cr1-Cre (*B6J.B6N(Cg)-Cx3cr1tm1.1(cre)Jung/J)*; RRID: IMSR_JAX:025524), and Il6ra^fl^ (*B6;SJL-Il6ratm1.1Drew/J*; RRID: IMSR_JAX:012944) mice were obtained from Jackson Labs and maintained in group housing in our lab. For tissue collection, mice were anesthetized with isoflurane prior to transcardial perfusion with 10 ml of 0.9% w/v sodium chloride solution followed by 25 ml of ice-cold 4% paraformaldehyde (PFA) in 0.9% w/v sodium chloride solution. Both male and female mice were analyzed in this study. Animals were included or excluded from the study based only on genotype and age. All experiments were performed under NIH guidelines and animal protocols approved by MGH/HMS (Protocol# 2023N000119) Institutional Animal Care and Use Committee.

#### Cell Lines

The IMG murine microglial cell line was obtained from Millipore (SCC134; RRID: CVCL_HC49). The sex of the original donor from which this cell line was established is not known. The BV2 murine microglial cell line was obtained from AcceGen (ABC-TC212S; RRID:CVCL_0182). The cells were cultured on uncoated 24- or 48-well plates in a humidified incubator at 37°C with 5% CO2 in DMEM-F12 supplemented with 10% fetal bovine serum (FBS) and 1% penicillin/streptomycin (PS).

The RAH-NSC line (primary rat adult hippocampal neural stem cells) is derived from young adult female Fisher 344 rats and was obtained from Millipore (#SCR022). The cells were cultured on 24- or 48-well plates coated with Poly-L-ornithine (Millipore #P3655) and Laminin (Millipore #CC095) in a humidified incubator at 37°C with 5% CO2 in Rat Neural Stem Cell Expansion Medium (Millipore #SCM009) containing DMEM/F12, basic fibroblast growth factor (bFGF), B27 supplement, 2 mM L-glutamine and a 1x solution of penicillin, streptomycin and fungizone (PSF).

### Methodology:

#### Transcardial Perfusion and Paraformaldehyde (PFA) Fixation

Mice were deeply anesthetized with isoflurane and perfused transcardially with 4% PFA in cold 0.1 M phosphate buffer (pH 7.4) after 0.9% NaCl. The brains were postfixed overnight and then transferred into 30% sucrose and kept there until fully saturated.

#### RNAscope

Coronal mouse brain sections were collected at 40μm thickness, washed, and baked onto microscope slides at 60°C for 30 minutes. All labeling procedures were performed using the Multiplex Fluorescent Reagent Kit v2 (Advanced Cell Diagnostics #323136) according to the manufacturer’s instructions. The following probes were used: Mm-Cx3cr1 (#314221), Mm-Gfap (#313211), Mm-Sox2 (#401041), Mm-Il6ra (#435921), and Mm-Il6 (#315891).

#### Immunohistochemistry

40 μm coronal mouse brain sections were collected on a microtome and blocked using 5% normal donkey serum (Sigma-Aldrich, #D9663–10ML) and 0.1% Triton X-100 in 1x phosphate-buffered saline (PBS) for a minimum of 30 minutes at room temperature. Sections were then incubated with primary antibodies overnight in blocking buffer. Next, sections were washed 3 times with PBS for 10 minutes each and subsequently incubated for 2 hours with Alexa Fluor fluorescently tagged secondary antibodies and DAPI. Sections were then washed 3 times with PBS for 10 minutes and mounted onto microscope slides with polyvinyl alcohol mounting medium with DABCO (PVA-DABCO). Primary antibodies used include: Rat anti-BrdU (1:100, Abcam #AB6326; RRID: AB_2313786), Mouse anti-NeuN (1:250, Sigma-Aldrich #MAB377; RRID:AB_2298772), Rabbit anti-Iba1 (1:500, FUJIFILM Wako Pure Chemical Corporation #019–19741; RRID:AB_839504), Mouse anti-Iba1 (1:250, (Thermo Fisher Scientific #MA5–27726; RRID:AB_2735228), Rabbit anti-Cleaved-caspase-3 (1:400, Cell Signaling Technology #9661; RRID:AB_2341188), Mouse IgG1 anti-doublecortin (1:100, Santa Cruz #sc-271390; RRID:AB_302459), Cell nuclei were counterstained with DAPI.

#### Immunocytochemistry

Immunocytochemistry was performed on IMG microglia and RAH-NSCs following fixation in multi-well cell culture plates. (4% PFA, 15 minutes). Cells were washed with 1x PBS and permeabilized with 0.1% Triton X-100 and blocked with 5% Donkey Serum. Primary antibodies used include: Chicken anti-GFAP (1:1,000, Abcam #AB134436, RRID:AB_2818977) and Mouse anti-Beta-Tubulin III (TUJ1) (1:100, R&D Systems #MAB1195, RRID:AB_357520). Cell nuclei were counterstained with DAPI.

#### Phagocytosis Assay

IMG microglia were plated at a density of 200,000 cells per well in 24-well plates and incubated overnight. The next day, cells were incubated for 24 hours with rIL-6, rIL-6Ra, H-IL-6, or sham. FluoSphere polystyrene microspheres, 1.0 μm diameter, yellow-green fluorescent (505/515) (Thermo Fisher Scientific #F13081) were rinsed in fetal bovine serum (FBS) for 5 minutes at 37C to reduce microsphere aggregation. Microspheres were resuspended in IMG cell culture media containing either rIL-6, rIL-6Ra, H-IL-6, or sham and added to the wells of plated microglia. After 3 hours, the cells were washed 1x with PBS and 1x with PBS and 3% trypan blue to quench the fluorescence of microspheres that were not phagocytosed by microglia. Cells were fixed for 30 minutes with 4% PFA for 30 minutes and washed 3x with PBS. Immunocytochemistry was performed as described above to label microglia. Confocal Zstacks were collected at 20x magnification with a 1 μm step-size to assess the internalization of microspheres by microglia. The number of fully internalized microspheres per microglia was counted and compared between groups.

#### Cytokine Array XL

We assayed the secretory profile of IMG microglia using the Proteome Profiler Mouse XL Cytokine Array (R&D Systems #ARY028) following the manufacturer’s instructions. Briefly, IMG microglia were cultured in uncoated 6-well plates with the minimum volume of media allowed by the culture plate and received a treatment or sham for 24 hours. All membranes were imaged simultaneously with a 10-minute chemiluminescent exposure using a Li-Cor Odyssey M. The intensity (mean gray value) of each spot on the membrane was quantified using Image J software.

#### ELISA

ELISAs for IL-6 (R&D Systems #M6000B), IL-6R (R&D Systems #MR600), Cx3cl1 (R&D Systems #MCX310), and CD200 (Aviva Biosystems #OKBB00526) were performed following the manufacturer’s instructions. Briefly, IMG microglia were cultured in uncoated 6-well plates with the minimum volume of media allowed by the culture plate and received a treatment or sham for 24 hours, after which the media was collected and used in the assay. Absorbance values were collected following 450 nM exposure and subtraction of background signal recorded following 570 nM exposure on a BioTek Synergy NEO2 multimode reader.

#### Confocal Microscopy

All images were taken with a Nikon C2 confocal light microscope with a 4x air objective (0.2 numerical aperture), 10x air objective (0.3 numerical aperture), 20x air objective (0.75 numerical aperture) or 40x oil immersion objective (1.3 numerical aperture) using the NIS-Elements software. Optical slices were captured at 1 μm intervals between each Z-limit of the sample being imaged. Images were collapsed into two-dimensional maximum intensity projections for analysis, unless otherwise noted.

#### Fluorescence Intensity Analysis

The intensity of GFAP and TUJ1 signal were assessed by outlining the somata of the cells of interest, separating each channel, and converting the channels to gray scale. The mean-gray value of each cell’s soma was recorded and compared between groups.

#### Nearest Neighbor Assay

The nearest neighbor analysis was carried out as described previously (29). Briefly, the linear distance from the center of each microglia soma to the center of the nearest microglia soma was measured. All linear measurements were made using the line tool in the ImageJ software. The nearest neighbor regularity index (NNRI) was calculated by dividing the average nearest neighbor distance by the standard deviation.

#### NSC Cluster Size

NSC cluster size was recorded by recording the area of clusters using the freehand tool in the ImageJ software.

#### Proximity Ring Analysis

To assess which and how many cells are proximal to a cell of interest, we centered and overlayed a circle with a 2,000 μm^2^ area on the cell of interest. The number and type of cells either fully or partially within the circle were counted as being within the proximity ring.

#### DCX Type 1-2-3 Assay

DCX+ cells in the SGZ of mice were binned into one of three groups as described previously (30). Briefly, type 1 cells have short or no processes, type 2 cells have medium length processes, and type 3 cells have large dendrites with branches that stretch into or fully through the GCL.

#### Microglia Morphology

Microglia soma size was recorded by outlining somata using the freehand tool in the ImageJ software and recording the area.

#### CD68 in Microglia

CD68 signal within microglia somata was outlined using the freehand tool in the ImageJ software and the area was recorded.

#### Cell Density

The density of cells in each well was calculated by counting the total number of DAPI+ nuclei visible within a single image.

#### BrdU Cell Proliferation

The in-vitro BrdU assay was carried out using the BrdU Cell Proliferation ELISA Kit (colorimetric) (Abcam #AB126556) according to the manufacturer’s instructions.

#### Cell Counting

NSCs were stained with antibodies against GFAP and TUJ1. Nuclei were counterstained with DAPI. 20x images were collected in four identical quadrants in each well. The number of cells per image were counted, averaged between quadrants, and compared between groups.

#### BrdU Injections

BrdU was dissolved in 0.9% NaCl at a concentration of 20 mg/ml and was filtered (0.2 μm) under sterile conditions. Mice received one intraperitoneal injection (100 mg/kg of body weight) 1 day or 1 month prior to sacrifice and were perfused and processed for BrdU immunohistochemistry to identify proliferating or surviving neural progenitor cells (NPCs), respectively. All mice received injections of the same preparation of BrdU at the same time.

#### Tissue Processing for BrdU Immunostaining

Mice were perfused as described above. 40 μm coronal sections were cut from a dry ice-cooled block on a sliding microtome (Leica, SM2010R). The sections were stored at 4°C in a cryoprotective buffer containing 28% ethylene glycol, 23% glycerin and 0.05 M phosphate buffer until the sections were processed for immunohistochemistry or immunofluorescence. DNA was denatured by incubating the sections for 2 hours in the 50% formamide/2× SSC (0.3 M NaCl and 0.03 M Sodium citrate) at 65°C.

Sections were rinsed for 15 min in 2× SSC and incubated for 30 min in 2 N HCl at 37°C. Acid was neutralized by rinsing the sections for 10 min in 0.1 M boric acid (pH 8.5) followed by several washes in PBS. The sections were blocked with PBS containing 0.1% Triton X-100/5% donkey serum, and then incubated with the primary antibodies overnight at 4°C. The sections were incubated with the secondary antibodies in PBS containing 0.1% Triton X-100 (TBS+) for 4 hours at room temperature protected from light. Sections were then washed with PBS+ and PBS, and then coverslipped in PVA-DABCO (Sigma, St. Louis, MO) as anti-fading agent.

#### RNA Sequencing

##### Extraction

Total RNA was extracted from fresh frozen tissue samples using Qiagen RNeasy Plus Universal mini kit following manufacturer’s instructions (Qiagen, Hilden, Germany).

##### Library Preparation with PolyA selection and Illumina Sequencing

RNA samples were quantified using Qubit 4 Fluorometer (Life Technologies, Carlsbad, CA, USA) and RNA integrity was checked using Agilent TapeStation 4200 (Agilent Technologies, Palo Alto, CA, USA). The NEBNext Ultra II RNA Library Prep Kit for Illumina & NEBNext Poly(A) mRNA Magnetic Isolation Module (New England Biolabs, Ipswich, MA, USA), including clustering and sequencing reagents, was utilized according to the manufacturer’s recommendations. Briefly, mRNAs were initially enriched with Oligod(T) beads. Enriched mRNAs were fragmented for 15 minutes at 94°C. First strand and second strand cDNA were subsequently synthesized. cDNA fragments were end repaired and adenylated at 3’ends, and universal adapters were ligated to cDNA fragments, followed by index addition and library enrichment by PCR with limited cycles. The sequencing library was validated on the Agilent TapeStation (Agilent Technologies, Palo Alto, CA, USA), and quantified by using Qubit 2.0 Fluorometer (ThermoFisher Scientific, Waltham, MA, USA) as well as by quantitative PCR (KAPA Biosystems, Wilmington, MA, USA).

##### Data Analysis

After investigating the quality of the raw data, sequence reads were trimmed to remove possible adapter sequences and nucleotides with poor quality using Trimmomatic v.0.36. The trimmed reads were mapped to the Rattus norvegicus Rnor6.0 reference genome available on ENSEMBL using the STAR aligner v.2.5.2b. BAM files were generated as a result of this step. Unique gene hit counts were calculated by using feature Counts from the Subread package v.1.5.2. Only unique reads that fell within exon regions were counted. After extraction of gene hit counts, the gene hit counts table was used for downstream differential expression analysis. Using DESeq2, a comparison of gene expression between the groups of samples was performed. The Wald test was used to generate P values and Log2 fold changes. GO terms were excluded from analysis if P>.05 or if fewer than 33% of the genes associated with that GO term were activated.

#### Conditioned Media Assays

Conditioned media was collected and either assayed directly or concentrated using Pierce Protein Concentrators with a 3K MWCO and 5 to 20 mL sample volume capacity according to the manufacturer’s instructions. Samples were centrifuged until concentrated 5x and subsequently added at a 1:5 ratio to fresh microglia cell culture media.

#### Efferocytosis Assay

IMG microglia were plated in uncoated cell culture dishes and grown to approximately 75% confluence. RAH-NSCs were plated in laminin and poly-L-ornithine-coated plates. After 24 hours, RAH-NSCs were treated with 100 μM staurosporine in NSC media for one hour to induce apoptosis. RAH-NSCs were then dissociated, counted, and plated at a ratio of 1:5 in the wells containing IMG microglia.

#### Contextual Fear Conditioning Assay

CFC assays were performed as previously described (33). Briefly Conditioning was conducted in two distinct contexts: context A with a shock, and context B with no shock. The test cage (17.8 × 17.8 × 30.5 cm) was encased by an isolation cubicle. Context A was composed of two plexiglass walls, two metal walls and a stainless-steel grid floor (Coulbourn Instruments). For context A, the white light was turned off and 70% ethanol was used for cleaning. For context B the white light was turned on and a peroxide-based cleaning solution was used. The shape and material of the chamber was also altered with a paper-based circular insert and flat flooring which was placed on top of the stainless-steel grid floor. Motion was recorded by a digital video camera mounted in front of the test cage. On day 0 mice were tested only in context A, then for 3 consecutive days in both A and B context with an hour separation. In test cage A the mice received a single 2 s foot shock (0.75 mA at the 185th second). Video Freeze software was used for recording freezing behavior. Freezing time during the first 180 s in each context for each day was recorded. Discrimination ratios were calculated as described previously (26).

### QUANTIFICATION AND STATISTICAL ANALYSIS

An unpaired Student’s t-test was used to compare 2 means. A one- or two-way ANOVA with Tukey’s post hoc analysis was used to compare 3 or more means. A one-way ANOVA with Dunnett’s post hoc analysis was used to compare 2 or more means to a control or sham condition. Information about the application of statistical analyses is listed in the figure legends. Results of statistical tests are reported in the text. Data are expressed as the mean ± standard error of the mean (SEM), and p < .05 was considered statistically significant. The number of replicates is as follows: Figure 1 (n=3), Figure 2 (n=4), Figure 2F–G (n=4, pooled), Figure 3 (n=4), Figure 4A–G (n=5, male), Figure 4H–O (n=4), Figure 5B–F’ (n=4, male), Figure 5G–H (n=4, male; n=4, female), Figure 5J–N (n=3 Cx3cr1-WT x IL-6R^fl/fl^ male, n=3 Cx3cr1-WT x IL-6R^fl/fl^ female, n=5 Cx3cr1-Cre x IL-6R^fl/fl^ male, n=3 Cx3cr1-Cre x IL-6R^fl/fl^ female).

GraphPad Prism (RRID: SCR_002798) was used for all statistical analyses.

## Supplementary Material

Supplementary Files

This is a list of supplementary files associated with this preprint. Click to download.

- CastroetalSupp.MaterialsNatureFormat.docx
- Graphicalabstract.docx

## Figures and Tables

**Table:** KEY RESOURCES TABLE

REAGENT or RESOURCE	SOURCE	IDENTIFIER
**Antibodies**		
Rat anti-BrdU	ABCAM	RRID: AB_2313786
Mouse anti-NeuN	Sigma-Aldrich	RRID: AB_2298772
Rabbit anti-Ibal	FUJIFILM Wako Pure Chemical Corporation	RRID: AB_839504
Mouse anti-Ibal	Thermo Fisher Scientific	RRID: AB_2735228
Rabbit anti-Cleaved-caspase-3	Cell Signaling Technology	RRID: AB_2341188
Mouse IgG1 anti-doublecortin	Santa Cruz Biotechnology	RRID: AB_10610966
Chicken anti-GFAP	ABCAM	RRID: AB_2818977
Mouse anti-Beta-Tubulin III (TUJ1)	R&D Systems	RRID: AB_357520
Rat anti-CD68	BIO-RAD	RRID: AB_869007
Donkey Anti-Rabbit IgG H&L (Alexa Fluor^®^ 488)	ABCAM	RRID: AB_2636877
Donkey Anti-Rabbit IgG H&L (Alexa Fluor^®^ 647)	ABCAM	RRID: AB_2752244
Donkey Anti-Mouse IgG H&L (Alexa Fluor^®^ 488)	Invitrogen	RRID: AB_141607
Donkey Anti-Mouse IgG H&L (Alexa Fluor^®^ 647)	ABCAM	RRID: AB_2890037
Donkey Anti-Rat IgG H&L (Alexa Fluor^®^ 568)	ABCAM	RRID: AB_2636887
Goat anti-Chicken IgY H&L (Alexa Fluor^®^ 568)	Invitrogen	RRID: AB_2534098
**Chemicals, peptides, and recombinant proteins**		
Recombinant Mouse IL-6 Protein	R&D Systems	CAT#: 406-ML025-CF
Recombinant Mouse IL-6R Alpha Protein	R&D Systems	CAT#: 1830-SR-025/CF
Recombinant Mouse IL-6/IL-6R Alpha Protein Chimera	R&D Systems	CAT#: 9038-SR-025/CF
**Critical commercial assays**		
Proteome Profiler Mouse XL Cytokine Array	R&D Systems	CAT#: ARY028
IL-6 ELISA Kit	R&D Systems	CAT#: M6000B
IL-6R ELISA Kit	R&D Systems	CAT#: MR600
CX3CL1 ELISA Kit	R&D Systems	CAT#: MCX310
CD200 ELISA Kit	AVIVA Biosystems	CAT#: OKBB00526
BrdU Cell Proliferation ELISA Kit	ABCAM	CAT#: AB126556
Multiplex Fluorescent Reagent Kit v2	Advanced Cell Diagnostics	CAT#: 323136
**Experimental models: Cell lines**		
Immortalized Murine Microglia Cells (IMG)	Millipore	RRID: CVCL_HC49
Immortalized Murine Microglia Cells (BV2)	AcceGen	RRID:CVCL_0182
Primary Rat Adult Hippocampal Neural Stem Cells	Millipore	CAT#: SCR022
**Experimental models: Organisms/strains**		
*C57BL/6J* Mice	Jackson Labs	RRID: IMSR_JAX:000664
*B6J.B6N(Cg)-Cx3crltml.l(cre)Jung/J* Mice	Jackson Labs	RRID: IMSR_JAX:025524
*B6;SJL-Il6ratm1.1Drew/J* Mice	Jackson Labs	RRID: IMSR_JAX:012944
**Software and algorithms**		
GraphPad Prism	Dotmatics	RRID: SCR_002798
**Other**		
RNAscope Probe Mm-Cx3cr1	Advanced Cell Diagnostics	CAT#: 314221
RNAscope Probe Mm-Gfap	Advanced Cell Diagnostics	CAT#: 313211
RNAscope Probe Mm-Sox2	Advanced Cell Diagnostics	CAT#: 401041
RNAscope Probe Mm-Il6ra	Advanced Cell Diagnostics	CAT#: 435921
RNAscope Probe Mm-Il6	Advanced Cell Diagnostics	CAT#: 315891
FluoSphere polystyrene microspheres, 1.0 μm diameter, yellow-green fluorescent (505/515)	Thermo Fisher Scientific	CAT#: F13081
BrdU (5-Bromo-2’-Deoxyuridine)	Millipore Sigma	CAT#: B5002
Polyvinyl alcohol mounting medium with DABCO^®^, antifading	Millipore Sigma	CAT#: 10981
